# Supplementary material for: MetaQTL: a package of new computational methods for the meta-analysis of QTL mapping experiments
Source: BMC Bioinformatics. 2007 Feb 8;8:49. doi: 10.1186/1471-2105-8-49 (PMC1808479; doi:10.1186/1471-2105-8-49)
Supplement: Additional File 7 — MetaQTL Package : jar file and tutorial. This Zip archive contains both the MetaQTL JAR file and the files of the tutorial. [file 1471-2105-8-49-S7.zip › org.inra.metaqtl/doc/org/metaqtl/graph/ChromLayer.html]

ChromLayer


|  |  |  |
| --- | --- | --- |
| **Overview** \| **Package** \| **Class** \| **Use** \| **Tree** \| **Deprecated** \| **Index** \| **Help** | |  |
| **PREV CLASS**   **NEXT CLASS** | **FRAMES**    **NO FRAMES**     **All Classes** |  |
| SUMMARY: NESTED \| FIELD \| CONSTR \| METHOD | DETAIL: FIELD \| CONSTR \| METHOD |  |


---


## org.metaqtl.graph Class ChromLayer

```
java.lang.Object
  org.metaqtl.graph.AnchoredElement
      org.metaqtl.graph.Layer
          org.metaqtl.graph.ChromLayer
```

---

``` public class ChromLayer extends Layer ```

---

| **Nested Class Summary** | |
| --- | --- |
| `class` | `ChromLayer.CMrkTickPath` |
| `class` | `ChromLayer.CMrkTickPoint` |


| **Field Summary** | |
| --- | --- |
| `static int` | `ALIGN_FIRST_COMMON` |
| `static int` | `ALIGN_FIRST_MARKER` |
| `static int` | `LEFT_LABEL` |
| `static int` | `NEG_COMMON` |
| `static int` | `POS_COMMON` |
| `static int` | `RIGHT_LABEL` |
| `static int` | `SINGLE_COMMON` |

**Fields inherited from class org.metaqtl.graph.Layer**

`height, width`

**Fields inherited from class org.metaqtl.graph.AnchoredElement**

`x, y`


**Constructor Summary**

`ChromLayer(double x,
double y)`


| **Method Summary** | |
| --- | --- |
| `static void` | `alignChromLayers(ChromLayer ref, ChromLayer layer, int mode)` |
| `void` | `attach(java.lang.Object object)`             Attach the given `object` to the layer. |
| `void` | `build(java.awt.Graphics2D graph)`             Build the layer. |
| `void` | `draw(java.awt.Graphics2D graph)`             Draws the layer on the given `graph` context. |
| `ChromAxe` | `getChromAxe()`             Return the axe of the chromosome. |
| `double` | `getChromWidth()` |
| `ChromLayer.CMrkTickPath[]` | `getCommonMarkerPaths(ChromLayer chromLayer)` |
| `ChromLayer.CMrkTickPoint[]` | `getCommonMarkerPoints(ChromLayer chromLayer)` |
| `double` | `getHeight()` |
| `java.awt.geom.Point2D` | `getMrkTickPoint2D(int mrkIdx, boolean reverse)` |
| `double` | `getWidth()` |
| `double` | `getYMax()` |
| `double` | `getYMin()` |
| `boolean` | `hasProba()` |
| `void` | `setLabelSide(int side)` |

**Methods inherited from class org.metaqtl.graph.AnchoredElement**

`getX, getY, setX, setY`

**Methods inherited from class java.lang.Object**

`clone, equals, finalize, getClass, hashCode, notify, notifyAll, toString, wait, wait, wait`

**Field Detail**

### RIGHT\_LABEL

```
public static final int RIGHT_LABEL
```

**See Also:**: Constant Field Values

---


### LEFT\_LABEL

```
public static final int LEFT_LABEL
```

**See Also:**: Constant Field Values

---


### ALIGN\_FIRST\_COMMON

```
public static final int ALIGN_FIRST_COMMON
```

**See Also:**: Constant Field Values

---


### ALIGN\_FIRST\_MARKER

```
public static final int ALIGN_FIRST_MARKER
```

**See Also:**: Constant Field Values

---


### SINGLE\_COMMON

```
public static final int SINGLE_COMMON
```

**See Also:**: Constant Field Values

---


### POS\_COMMON

```
public static final int POS_COMMON
```

**See Also:**: Constant Field Values

---


### NEG\_COMMON

```
public static final int NEG_COMMON
```

**See Also:**: Constant Field Values


**Constructor Detail**

### ChromLayer

```
public ChromLayer(double x,
                  double y)
```

**Parameters:**: `x` -: `y` -


**Method Detail**

### alignChromLayers

```
public static void alignChromLayers(ChromLayer ref,
                                    ChromLayer layer,
                                    int mode)
```

:   **Parameters:**: `ref` -: `layer` -: `mode` -

---


### draw

```
public void draw(java.awt.Graphics2D graph)
```

:   **Description copied from class: `Layer`**
:   Draws the layer on the given `graph` context.

    :   **Specified by:**: `draw` in class `Layer`

---


### build

```
public void build(java.awt.Graphics2D graph)
```

:   **Description copied from class: `Layer`**
:   Build the layer.

    :   **Specified by:**: `build` in class `Layer`

---


### getChromAxe

```
public ChromAxe getChromAxe()
```

:   Return the axe of the chromosome.

    :   **Returns:**

---


### attach

```
public void attach(java.lang.Object object)
```

:   **Description copied from class: `Layer`**
:   Attach the given `object` to the layer.

    :   **Specified by:**: `attach` in class `Layer`
    :   **Parameters:**: `object` - the object to attach to the layer.

---


### setLabelSide

```
public void setLabelSide(int side)
```

---


### getHeight

```
public double getHeight()
```

:   **Overrides:**: `getHeight` in class `Layer`
:   **Returns:**: Returns the height.

---


### getWidth

```
public double getWidth()
```

:   **Overrides:**: `getWidth` in class `Layer`
:   **Returns:**: Returns the width.

---


### getYMin

```
public double getYMin()
```

:   **Specified by:**: `getYMin` in class `Layer`

---


### getYMax

```
public double getYMax()
```

:   **Specified by:**: `getYMax` in class `Layer`

---


### getCommonMarkerPoints

```
public ChromLayer.CMrkTickPoint[] getCommonMarkerPoints(ChromLayer chromLayer)
```

:   **Parameters:**: `chromLayer` -

---


### getCommonMarkerPaths

```
public ChromLayer.CMrkTickPath[] getCommonMarkerPaths(ChromLayer chromLayer)
```

---


### getChromWidth

```
public double getChromWidth()
```

---


### getMrkTickPoint2D

```
public java.awt.geom.Point2D getMrkTickPoint2D(int mrkIdx,
                                               boolean reverse)
```

---


### hasProba

```
public boolean hasProba()
```

:   **Returns:**


---


|  |  |  |
| --- | --- | --- |
| **Overview** \| **Package** \| **Class** \| **Use** \| **Tree** \| **Deprecated** \| **Index** \| **Help** | |  |
| **PREV CLASS**   **NEXT CLASS** | **FRAMES**    **NO FRAMES**     **All Classes** |  |
| SUMMARY: NESTED \| FIELD \| CONSTR \| METHOD | DETAIL: FIELD \| CONSTR \| METHOD |  |


---
